# Stroke in patients with left ventricular assist device (LVAD): who is at risk?—a retrospective observational study at a tertiary care center

**DOI:** 10.3389/fcvm.2025.1591208

**Published:** 2025-09-09

**Authors:** Yasemin Akalan, Hans Worthmann, Dominik Berliner, Juliane Hupe, Gerrit M. Grosse, Omar Abu-Fares, Kim K. Ravenberg, Karin Weissenborn, Arjang Ruhparwar, Steven R. Talbot, Johann Bauersachs, Jan D. Schmitto, Jasmin S. Hanke, Maria M. Gabriel

**Affiliations:** ^1^ Department of Cardiology and Angiology, Hannover Medical School, Hannover, Germany; ^2^ Department of Neurology, Hannover Medical School, Hannover, Germany; ^3^ Department of Vascular and Endovascular Surgery, Klinikum Wolfsburg, Wolfsburg, Germany; ^4^ Department of Neurology and Stroke Center, University Hospital Basel, Basel, Switzerland; ^5^ Institute of Diagnostic and Interventional Neuroradiology, Hannover Medical School, Hannover, Germany; ^6^ Department of Radiology, Klinikum Osnabrueck, Osnabrueck, Germany; ^7^ Department of Cardiac, Thoracic, Transplantation and Vascular Surgery, Hannover Medical School, Hannover, Germany; ^8^ Institute for Laboratory Animal Science, Hannover Medical School, Hannover, Germany

**Keywords:** ischemic stroke, intracranial bleeding, HeartMate 3, HeartWare, left ventricular assist device, LVAD, mechanical circulatory support, hemorrhage

## Abstract

**Objectives:**

Stroke is a severe complication in patients with left ventricular assist devices (LVAD), significantly affecting quality of life and potentially leading to death. This study aimed to illustrate the clinical features, outcomes, and risk factors associated with stroke in LVAD patients, with the goal of identifying potential treatment targets.

**Methods:**

In a study of 249 consecutive patients who underwent LVAD implantation, detailed evaluations were conducted regarding clinical characteristics, perioperative management, cardiovascular risk factors, comorbidities, and brain imaging. The etiology, treatment, and outcomes were subsequently assessed in individuals who encountered a stroke.

**Results:**

Eighty-three cerebrovascular events (CVE) occurred in 54/249 patients during a median study period of 2.2 years (0.4–3.5) with 53 ischemic events and 22 intracranial hemorrhages (ICH). Early peri- or postoperatively CVE in context to the LVAD implantation were identified in 31 patients. Competing risks regression analysis revealed that postoperative dialysis was associated with higher risk for CVE, considering death as competing risk event (HR 3.617; 95%-CI: 1.78–7.35; *p* ≤ 0.001). Modified Rankin Scale at outpatient visit did not differ in early CVE [3 (IQR 2–5) vs. 3 (IQR2–4), *p* = 0.146]. Late CVE frequently occurred during hospitalization for sepsis or in cardiac rehabilitation [*n* = 16/41 events (39%)]. Competing risk analysis treating death and heart transplantation as competitors identified history of stroke as associated factor [HR 3.564; 95%-CI (1.67–7.169); *p* = 0.001]. Mortality was not associated with CVE [with *n* = 27/54 (50%) vs. without CVE 94/195 (48.2%) *p* = 0.183].

**Conclusion:**

Patients who require postoperative dialysis face a heightened risk for early cerebrovascular events (CVE) during and after LVAD implantation. Additionally, a history of stroke and complicated clinical courses should increase awareness regarding the potential for impending CVE in the long term.

## Introduction

Pathologies of the cardiovascular system accounted for nearly three million hospital admissions, making them the most common reason for hospitalization in Germany (aside from COVID). Additionally, with more than 330,000 deaths, cardiovascular diseases remain the most common cause of death (1). Due to the anticipated demographic changes in Western countries, the rate of severe heart failure is expected to rise even further. Patients diagnosed with terminal heart failure who do not respond to medical treatment should be considered for orthotopic heart transplantation, according to European guidelines (2). However, the number of available donor hearts remains low and does not meet the current demand (3).

Due to continuous technological improvements in the past decade, left ventricular assist devices (LVAD) are a viable option for patients who are in need of urgent therapy as well as for those who are not eligible for cardiac transplantation (4). Yet, LVAD therapy faces various challenges of accompanying hemocompatibility-related complications such as ischemic and hemorrhagic stroke (5, 6). Their occurrence is associated with increased mortality (7) and poor functional outcomes in survivors (5, 8).

Within the last decade, growing surgical and medical experience, as well as technological improvements, have contributed to the reduction of cerebrovascular events and improved outcomes for LVAD patients (9–13). According to the MOMENTUM 3 Trial, the implantation of a fully magnetically levitated centrifugal-flow LVAD (HeartMate3) was superior with respect to survival, free of disabling stroke or reoperation to replace or remove a malfunctioning device compared to axial-flow pumps (14).

The MOMENTUM 3 Trial highlights that stroke remains a significant concern even with the latest generation of left ventricular assist devices: During the 24-month follow-up period, 149 out of 1,028 patients experienced a stroke, with 78 of those identified as disabling strokes (13). Additionally, a total of 201 deaths were registered, of which 42 were directly attributable to stroke-related complications (14). Nonetheless, prolonged survival on LVAD therapy has shifted attention to challenges of the long-term management, including cerebrovascular pathologies (15, 16) demonstrating the ongoing risk of ischemic and hemorrhagic strokes (17–22). However, the pathomechanism may differ from those seen in early-onset stroke (20, 21) and may include different risk factors. Thus, CVE occurring in the peri-interventional or the first postoperative phase contrast those in the later stage (23, 24), which raises the question of differing risk factors in both groups.

New pathophysiological aspects must be continuously evaluated, and personalized approaches need to be consistently applied—particularly with regard to an old growing Bridge to Destination (BTD) population with additional cerebrovascular issues and, thus, even more complex homeostasis under LVAD treatment (16).

Current international registries on LVAD patients do not emphasize CVE details and lack analysis of specific risk factors or stroke-related clinical courses and outcomes (5). Therefore, this study aims to provide an updated survey on the prevalence of CVE in LVAD patients based on real-world data and to address the lack of analyses of specific stroke risk factors and stroke-related clinical courses in this group. Further insights might help to identify risk factors and raise awareness among medical teams for this unique and vulnerable population.

## Material and methods

This retrospective, monocentric study included 249 consecutive patients who received an LVAD between January 2015 and August 2020 at our high-volume heart failure clinic. All types of LVAD device were enrolled into the study. Patient data was reviewed between November 2020 and July 2021 for the occurrence of ischemic stroke or intracranial hemorrhage (ICH), the latter including subarachnoid, intraparenchymal, subdural, and epidural hemorrhages. Patients implanted in August 2020 were additionally resurveyed in August 2022 to ensure a minimum of two years of follow-up. Medical documentation was revised, including all available cranial imaging data. Clinical data, including medical findings and all available computed tomography (CT) scans of the head were screened by two specialized medical experts experienced in neurology (MMG) and cardiology (YA). Atypical or unclear CT scan findings were re-evaluated by a board-certified neuroradiologist (OAF). The diagnosis of ischemic cerebral infarction or ICH was made in case of an acute neurologic deficit and an according CT scan finding. In the case of incidentally detected hypodense lesions in CT scans not clearly attributable to clinical deficiencies and a lack of previous imaging for comparison, these events were declared “indeterminable” and not considered for further analysis. Timing, type, clinical severity, etiology and outcome of CVE, cardiovascular risk factors, concomitant diseases, information on surgical approaches and complications, laboratory data, coagulation management, and parameters of mechanical circulatory support were assessed. The severity of heart failure was classified according to the Interagency Registry for Mechanical Assisted Circulatory Support (INTERMACS) (25). Cardiovascular burden was quantified using the CHA2DS2-VASc score (26). Sepsis was defined and diagnosed according to the guidelines of the German Sepsis Society (27).

Clinical outcome was classified with the modified Rankin Scale (mRS) at the time of hospital discharge after LVAD implantation and after stroke, ranging from 0 with no functional impairment to 6, representing death (28). The National Institutes of Health Stroke Scale (NIHSS) was obtained directly if a neurologist had seen the patient at the time of stroke. In case of missing documentation, NIHSS was retrospectively scored regarding patients’ documented deficits at the stroke and was also elaborated regarding the final examination results at discharge. “Early stroke” was—in accordance with prior studies (29, 30)—defined as any cerebrovascular event (CVE) in context to the index hospitalization of LVAD implantation, including perioperative and postoperative strokes. “Perioperative stroke” was defined as any new clinical diagnosis of CVE within the first seven days post LVAD implantation or exchange. “Postoperative stroke” was defined as all strokes occurring from the 8th day until discharge. All CVE detected in a later follow-up after the first hospitalization was defined as “late stroke”. Further information are mentioned in the Supplementary Material S1.

All patients gave written informed consent for data analysis. The study was conducted following the Declaration of Helsinki and was approved by the local ethics committee (ethics vote no.: 2204-2014 and 2997-2016).

### Statistics

Statistical analysis and figures were created using SPSS © Statistics, Version 28 (©1989, 2021 by SPSS Inc., Chicago, Illinois, USA). Patient groups were classified according to the presence or absence of diagnosed stroke. The Kolmogorov–Smirnov test was used to test against the hypothesis of normal distribution. The median and interquartile ranges were reported for all non-normally distributed continuous values, and the Mann–Whitney *U* test was applied. A competing risks analysis was conducted using a Fine and Gray model to assess the cumulative incidence of stroke in two models: (a) early stroke, treating death as a competitor, and (b) late stroke, treating death and heart transplantation as competitors, respectively. Based on their end-of-survey time—defined as the date of data collection- the data were censored for patients who neither suffered a stroke nor died. Time-to-event data, including the interval between implantation and either follow-up or event occurrence (early stroke, late stroke, heart transplantation, or death), were analyzed using the “cmprsk” package in the R software (v4.4.0) (31). For each covariate, a competing risks regression model was fitted using the crr function, with time-to-event as the response variable, including censored patients and the factorized status as the competing event. The cumulative incidence of events across covariate groups was estimated using the “cuminc” function. The cumulative incidence functions were visualized using ggplot2 for all cofactors with significant associations (32). A summary of the model estimates was compiled, including hazard ratios (HR), 95%-confidence intervals (CI), and *p*-values. Wald tests were performed to evaluate the covariate's influence on the likelihood of each event. If competing risk factors were not needed to be considered, Pearson's chi-square test was used for group differences in categorical variables. *P*-values <0.05 were considered significant.

## Results

Median days on device during the entire study period was 819 days (157.5–1288) or 2.2 years (0.4–3.5) considering the interval between day of LVAD implantation and end-of-survey, death, or orthotopic heart transplantation. HeartWare HVAD (Johnson & Johnson, U.S.A.) was received by 120 patients (48.2%), while HeartMate 3 (Abbott, U.S.A.) was implanted in 119 patients (47.8%). Additionally, nine patients (3.6%) were implanted with HeartMate II (Abbott, U.S.A.) and one with a ReliantHeart aVAD (ReliantHeart Inc, U.S.A.). Overall, 27% (*n* = 67) of the study cohort had at least one cranial imaging after LVAD implantation. Less than half of the patients with CT scans underwent an additional contrast-enhanced angiography (*n* = 26; 10.4%).

Overall, 83 CVE were recorded in 54 (21.7%) of 249 patients on LVAD therapy, corresponding to 0.15 CVE per patient-year within the above-mentioned follow-up interval in all patients. Of note, the rate of 0.15 events per patient-years was also recorded when only HeartMate 3 patients were analyzed.

**Figure 1:**
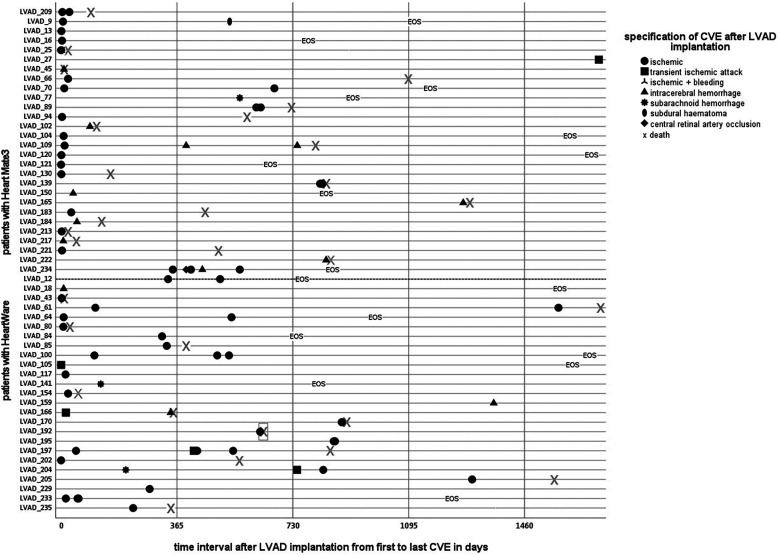
Time course of cerebrovascular events after LVAD implantation. Patients on LVAD therapy with cerebrovascular events. Individual courses with an illustration of each specific CVE, categorized into device groups by a dotted line: Heart Mate 3 on the upper, Heart Ware patients on the lower side, except the first patient (ID 209) on HeartMate II. A clustered occurrence, especially of ischemic events in the vulnerable postoperative phase, is apparent.

ICH occurred in 22 patients (intraparenchymal hemorrhage *n* = 18; subdural hematoma *n* = 1; subarachnoid hemorrhage *n* = 3), whereas most events were ischemic strokes (ischemic stroke (*n* = 46) and transient ischemic attacks (*n* = 7). In eight patients, indeterminable ischemic lesions were found incidentally in screening CT scans, but a concrete association with LVAD therapy was not possible. Figure 1 illustrates the chronological onset of CVEs and the follow-up course of all 54 patients affected by CVE. Of note, 21 patients (8%) suffered from recurrent strokes.

### Early stroke

During the hospitalization for the index LVAD implantation, 31 patients (12.4%) experienced 34 CVE, with the majority being ischemic (ischemic stroke *n* = 23, TIA *n* = 3, ICH *n* = 8). Their baseline characteristics are presented in Table 1. Perioperative CVE were registered in 16 cases, in median two days after implantation (IQR 0.25–3.75 days). Postoperative CVE were detected 22 days in median after LVAD implantation (IQR 10–38 days). Most early ischemic events were in the anterior circulation (*n* = 19; 82.6%), and only a few in the posterior circulation (*n* = 4; 17.4%). Cardiac thrombotic material was perioperatively identified in nine patients, showing a risk for early strokes [*n* = 3/31 (9.7%) vs. *n* = 6/218 (2.8%), *p* = 0.029].

**Table 1:** Baseline clinical characteristics of LVAD patients with and without early strokes after LVAD implantation and competing risk regression analysis.

Characteristics	Patients with early stroke *n* = 31	Patients without early stroke *n* = 218	Hazard ratio	Standard error	95%-confidence interval	*P*- value
Female	2 (6.5%)	24 (11%)	0.583	0.733	0.139–2.455	0.46
Age at implantation	57 (50–66)	59 (51–66)				0.553
Device at 1. Implantation			1.835	0.376	0.879–3.833	0.11
HeartWare	11 (35.5%)	109 (50%)				
HeartMate3	19 (61.3%)	100 (45.9%)				
Minimal invasive surgery	11 (35.5%)	107 (49%)	1.699	0.372	0.82–3.522	0.15
Cardiomyopathy			1.271	0.356	0.632–2.556	0.5
ICM	16 (51.6%)	99 (45.4%)				
DCM	15 (48.4%)	110 (50.5%)				
Diabetes mellitus	11 (35.5%)	64 (28.9%)	1.278	0.371	0.618–2.642	0.51
Coronary heart disease	21 (67.7%)	130 (59.6%)	1.38	0.379	0.656–2.902	0.4
History of smoking	15 (48.4%)	89 (40.8%)	0.918	0.22	0.596–1.412	0.7
Alcohol intake	2 (6.5%)	16 (7.3%)	0.918	0.39	0.427–1.974	0.83
Arterial hypertension	15 (48.4%)	123 (56.4%)	0.723	0.357	0.359–1.455	0.36
Chronic kidney disease	13 (41.9%)	104 (47.7%)	0.792	0.361	0.39–1.609	0.52
Dialysis before implantation	5 (16.1%)	26 (11.9%)	1.438	0.483	0.559–3.703	0.45
Dialysis after implantation	18 (58%)	57 (26.1%)	3.617	0.362	1.78–7.35	**<0**.**001**
Stroke before LVAD	9 (29%)	41 (18.8%)	1.719	0.39	0.8–3.693	0.16
INTERMACS			0.996	0.238	0.625–1.587	0.99
INTERMACS 1	6 (19.4%)	41 (18.8%)				
INTERMACS 2	3 (9.7%)	21 (9.6%)				
INTERMACS 3	5 (16.1%)	40 (18.3%)				
INTERMACS 4	8 (25.8%)	54 (24.8%)				
INTERMACS 5	8 (25.8%)	48 (22%)				
INTERMACS 6	1 (3.2%)	14 (6.4%)				
INTERMACS 7	0	0				
Chad2Vas2-Score	2 (2–5)	3 (2–5)				0.406
AF	19 (61%)	133 (61%)	0.971	0.368	0.472–1.996	0.94

Early stroke is defined as any cerebrovascular event (CVE) in context to the index hospitalization of LVAD implantation, including perioperative and postoperative strokes, respectively. As indicated, data are presented as numbers (%) or median (IQR). A competing risk analysis was conducted using the Fine and Gray model to assess the cumulative incidence of early stroke with death as a competitor. Data was censored for all patients without early stroke or death at the end of the survey. Age and CHAD2VAS2-Score were analyzed using the Mann–Whitney-*U*-Test. Statistically significant results are shown in bold. *P* < 0.05 is considered statistically significant.

INTERMACS, interagency registry for mechanically assisted circulatory support; ICM, ischemic cardiomyopathy; DCM, dilatative cardiomyopathy; AF, atrial fibrillation.

**Figure 2:**
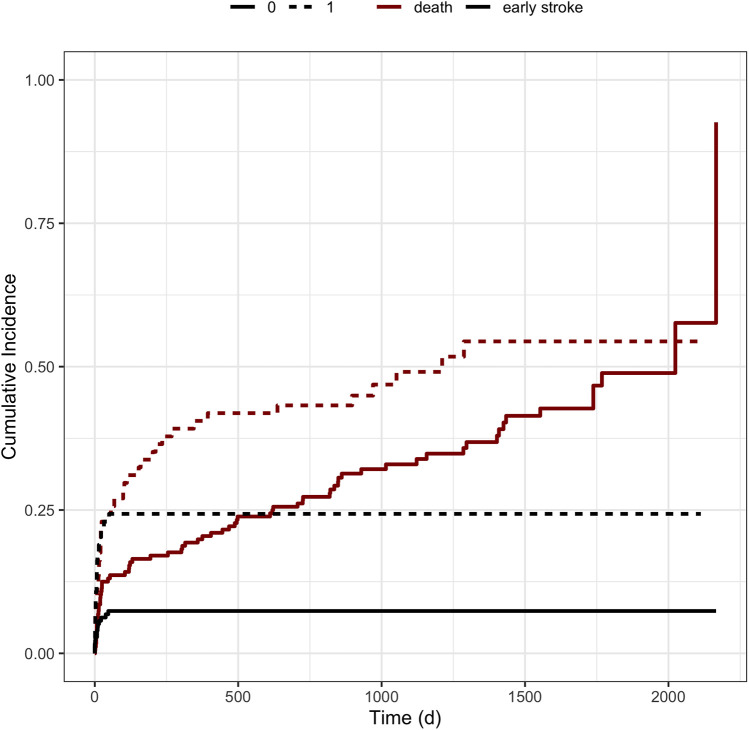
Cumulative incidence functions stratified by the need for dialysis. Figure illustrates the cumulative incidence functions stratified by the need for dialysis after LVAD implantation. The black lines represent the incidence of early strokes after LVAD implantation, whereas the red lines correspond to patients’ survival according to the covariates. Dotted lines characterize the need for dialysis, whereas solid lines represent the absence of postoperative dialysis.

The competing risks regression analysis revealed that the need for dialysis after LVAD implantation was associated with a significantly increased risk of early stroke or death (HR 1.286; 95%-CI: 1.78–7.35; *p* ≤ 0.0001) (Figure 2). Of note, patients with acute kidney failure before LVAD implantation with a need for dialysis did not suffer significantly more often from early stroke (*n* = 31, HR1.438; 95%-CI: 0.559–3.703; *p* = 0.45) (Table 1). Regarding the likelihood of early stroke or death, Wald tests revealed a test statistic of 13.85 and a *p*-value of <0.001 for the occurrence of early stroke and a test statistic of 4.16 with a *p*-value of 0.04 regarding the risk of death. The implantation of the HeartWare device, however, only showed a trend for an increased risk for early stroke compared to a HeartMate3 implantation, treating death as a competing risk event. (HR 1.835 (95%-CI: 0.879; 3.833; *p* = 0.11). Additionally, the type of surgical approach with regard to a conventional thoracotomy compared to a minimally invasive approach also revealed an increased risk for the development of early stroke with a competing risk for death (HR 1.699; 95%-CI: 0.82–3.522; *p* = 0.15).

Interestingly, at the time of peri- and postoperative stroke, in one-third (*n* = 11/34, 32.3%) of the cases, the criteria for sepsis was met in most of the patients driven by pneumonia (*n* = 9). Further characteristics such as preceding cardiogenic shock (*n* = 7/34), need for extracorporeal membrane oxygenation (ECMO) before implantation (*n* = 6/34), or prior Impella acute mechanical circulatory support device (*n* = 5/34) had no impact on the occurrence of early strokes or death. Only a few patients with early CVE showed further strokes in the long run (*n* = 7).

### Anticoagulation in patients with early stroke after LVAD implantation

Intracranial hemorrhages were identified under the continuous administration of heparin in 3 of 8 patients, whereas 3 occurred when argatroban was replaced by heparin following a negative heparin-induced thrombocytopenia test. Among patients with ICH, only one patient was simultaneously on antiplatelet therapy using acetylsalicylic acid. In two patients, heparin was administered and overlapped with phenprocoumon, with one patient experiencing an ICH with an INR within and another exceeding the intended therapeutic INR range.

All patients with early ischemic events (*n* = 31) were on heparin, but two were on argatroban. In five patients with ischemic CVE, a vitamin K antagonist (VKA) was administered in addition to heparin as bridging therapy. Three of these 5 had reached the therapeutic INR range. Among ischemic stroke patients, acetylsalicylic acid or clopidogrel, respectively, were administered in 11 of 31 patients in addition to heparin or VKA.

### Late stroke

In total, 41 late strokes were recorded in 31 patients (12.4%), in median 531 days (IQR 328–824 days) after LVAD implantation. Their baseline characteristics and, according to competing risks analysis, treating death and heart transplantation as competitors are summarized in Table 2. Of note, a considerable proportion of the total cohort died early after LVAD implantation (*n* = 51 within the first 100 days after LVAD implantation). It could thus not be followed up regarding cerebrovascular events (Figures 1, 4A).

**Table 2:** Baseline clinical characteristics of patients with and without late stroke after LVAD implantation and competing risk regression analysis.

Characteristics	Patients with late stroke *n* = 31	Patients without late stroke *n* = 218	Hazard ratio	Standard error	95%-confidence interval	*P*-value
Female	1 (3.2%)	25 (11.5%)	0.332	1.029	0.044–2.498	0.28
Age at implantation	55 (51–61)	59 (51–66)				0.553
Device at 1. implantation			0.695	0.389	0.324–1.49	0.35
HeartWare	18 (58.1%)	102 (46.8%)				
HeartMate3	13 (41.9%)	106 (48.6%)				
Minimal invasive surgery	16 (51.6%)	102 (46.8%)	1.176	0.383	0.554–2.493	0.67
Device exchange	7 (22.6%)	16 (7.3%)	2.324	0.502	0.869–6.216	0.093
Cardiomyopathy			0.831	0.388	0.388–1.778	0.63
ICM	13 (41.9%)	102 (46.8%)				
DCM	15 (48.4%)	110 (50.5%)				
Diabetes mellitus	8 (25.8%)	67 (30.7%)	0.817	0.434	0.349–1.912	0.64
Coronary heart disease	19 (61.3%)	132 (60.6%)	0.95	0.388	0.444–2.031	0.89
History of smoking	15 (48.4%)	89 (40.8%)	1.531	0.385	0.72–3.256	0.27
Alcohol intake	6 (19.4%)	12 (5.5%)	2.46	0.472	0.976–6.202	0.056
Arterial hypertension	20 (64.5%)	118 (54.1%)	1.945	0.427	0.843–4.491	0.12
Chronic kidney disease	18 (58.1%)	99 (45.4%)	1.384	0.384	0.652–2.936	0.4
Dialysis before implantation	5 (16.1%)	26 (11.9%)	1.385	0.538	0.482–3.975	0.55
Dialysis after implantation	9 (29.0%)	66 (30.3%)	0.824	0.439	0.349–1.947	0.66
Stroke before LVAD	13 (41.9%)	37 (17%)	3.762	0.383	1.776–7.969	**<0**.**001**
INTERMACS			2.891	0.395	1.333–6.268	**0**.**007**
INTERMACS 1	2 (6.7%)	45 (20.5%)				
INTERMACS 2	1 (3.3%)	23 (10.5%)				
INTERMACS 3	4 (13.3%)	41 (18.7%)				
INTERMACS 4	10 (33.3%)	51 (23.7%)				
INTERMACS 5	12 (40%)	44 (20.1%)				
INTERMACS 6	1 (3.3%)	14 (6.4%)				
INTERMACS 7	0	0				
Modified Ranking scale after implant						0.008
mRS 0	0	0				
mRS 1	4 (13.3%)	34 (15.5%)				
mRS 2	15 (50%)	59 (26.9%)				
mRS 3	10 (33.3%)	46 (21.3%)				
mRS 4	1 (3.3%)	17 (7.8%)				
mRS 5	0	62 (28.3%)				
Chad2Vas2-Score	3 (2–4)	3 (2–5)				0.406
AF	22 (73.3%)	130 (59.4%)	1.843	0.437	0.783–4.337	0.16

This table contains characteristics of patients suffering ischemic or hemorrhagic strokes after LVAD implantation in the long run- defined as strokes occurring after the index hospitalization when LVAD implantation was performed. As indicated, data are presented as numbers (%) or median (IQR). A competing risk analysis was conducted using the Fine and Gray model to assess the cumulative incidence of late stroke with death and heart transplantation as a competitor. Data was censored for all patients without late stroke, death, or heart transplantation at the end of the survey. Age, mRS and CHAD2VAS2 Score were analyzed using the Mann–Whitney-*U*-Test. Statistically significant results are shown in bold. *P* < 0.05 is considered statistically significant. ICM, ischemic cardiomyopathy; DCM, dilated cardiomyopathy; INTERMACS, interagency registry for mechanically assisted circulatory support; mRS, modified rankin scale; AF, atrial fibrillation.

Ischemic events occurred more often than cerebral bleeding (ischemic stroke *n* = 23, transient ischemic attack *n* = 4). Fourteen events were of hemorrhagic origin (intraparenchymal hemorrhage *n* = 10, subarachnoid hemorrhage *n* = 3, subdural hematoma *n* = 1). It is noteworthy that in five additional cases, incidentally ischemic stroke signs were found in imaging, which occurred after LVAD implantation—not corresponding to acute neurological symptoms and thus being considered as silent strokes.

**Figure 3:**
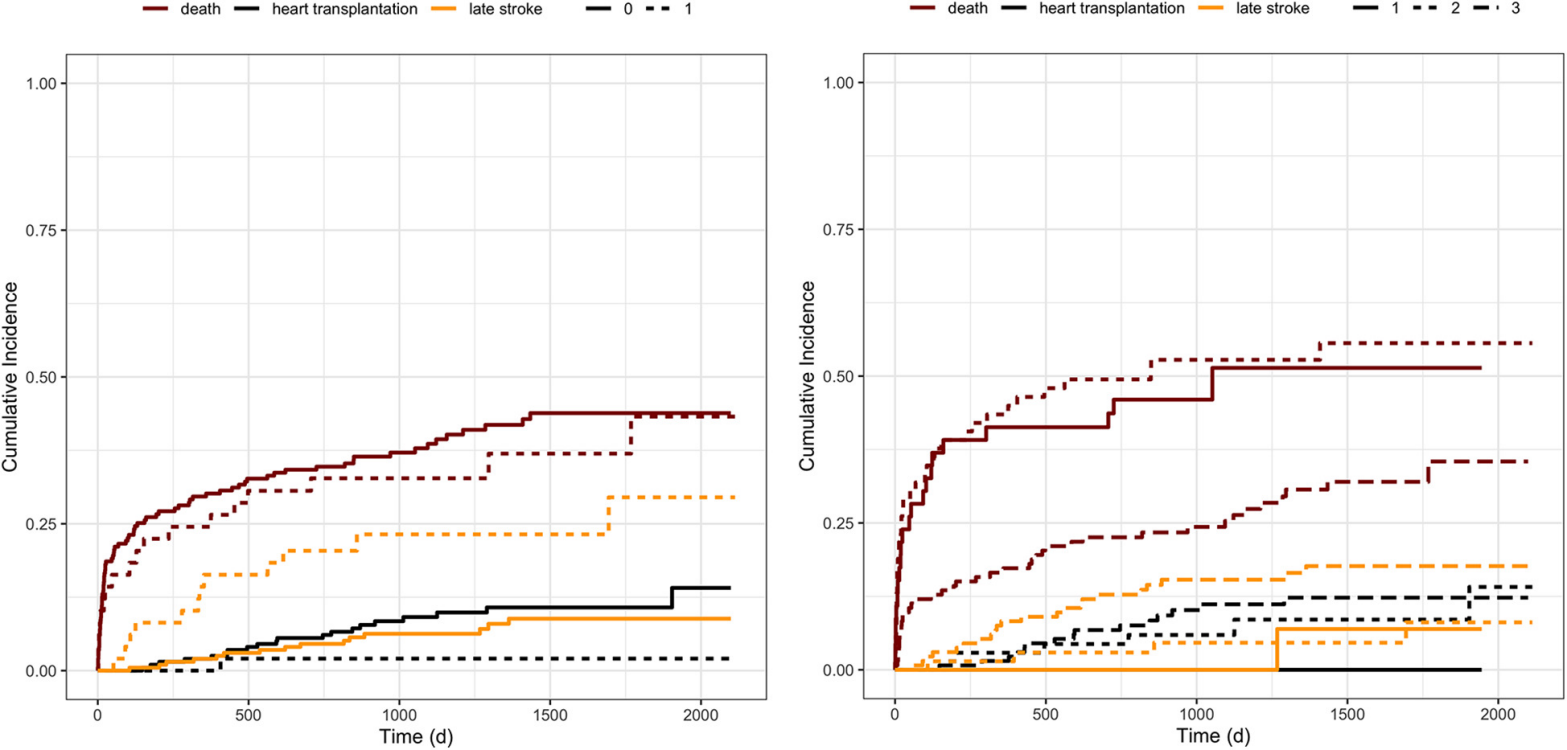
Cumulative incidence functions stratified by covariates. Figure illustrates the cumulative incidence functions of stroke, death, or heart transplantation stratified by left: history of stroke before LVAD implantation and right: the incidence according to the INTERMACS category at the time of implantation. The categories range from one, representing patients in severe cardiogenic shock, to seven, including heart failure patients with only a few functional deficits. The seven levels were categorized into 3 groups, summarizing INTERMACS level four to seven to barely, INTERMACS two and three to moderately, and INTERMACS one to severely impaired patients. The orange lines represent the incidence of late strokes, whereas the red lines correspond to patients’ survival, and the black line to heart transplantation. Left: solid lines represent patients without a history of stroke, and dotted lines, respectively, characterize the patients with a history of stroke. Right: solid lines represent INTERMACS level one, the dotted line INTERMACS level two and three, and the irregularly dotted line INTERMACS level four to seven.

Competing risks regression analysis revealed that a history of stroke before LVAD implantation was significantly associated with the risk for both, late hemorrhagic and late ischemic events, treating death or heart transplantation as competing risk factors [HR 3.564; 95%-CI (1.67–7.169); *p* = 0.001] (Figure 3 left). When evaluating the covariate's influence on the likelihood of each event, a history of stroke did not affect death (test statistic = 0.244, *p* = 0.620). Interestingly, cardiovascular risk factors such as AF, peripheral artery disease, arterial hypertension, and the history or current intake of alcohol revealed a trend towards a higher risk for late strokes without reaching the level of significance. Furthermore, there was a trend for patients needing device exchange and patients with a history of a cardiovascular bypass operation.

Patients with higher INTERMACS categories, and thus better preoperative conditions at the time of LVAD implantation, suffered more often from late strokes, seen that cumulative survival on LVAD therapy was significantly less in critically affected patients with low INTERMACS level [HR 2.891; 95% CI (1.333–6.268); *p* = 0.0072] (Figure 3 right).

Late CVE occurred often during hospitalization for acute complications (14/41). Reasons for hospitalization were frequently based on well-known complications during LVAD therapy: gastrointestinal bleeding (*n* = 2), sepsis (*n* = 6), LVAD thrombosis (*n* = 3), cardiac decompensation (*n* = 1), ileostomy placement (*n* = 1), INR derangement (*n* = 1). Among the patients with sepsis (*n* = 6), five had acute sepsis, and one had a chronic driveline infection. Two patients were admitted to rehabilitation centers while suffering from stroke.

### Anticoagulation in patients with late strokes after LVAD implantation

In the context of hospitalization, two patients were anticoagulated with argatroban and ten with heparin at the time of stroke occurrence. INR derangement is one of the most critical reported reasons for CVE in LVAD patients, and antiplatelet therapy in the context of late-occurring CVE are shown in Table 3. Regarding late-onset ischemic events, 10 did not occur in the context of hospitalization and were seen under VKA therapy. Only 3 of these 10 cases revealed inadequate INR values at the time of the first possible assessment after the onset of stroke symptoms. An antiplatelet therapy was registered in about half of the cases. Intracranial hemorrhages were considerably more frequent than ischemic events while on heparin (heparin at the time of intracranial hemorrhage *n* = 7/14, heparin or argatroban at the time of ischemic event *n* = 5/27). At the same time, antiplatelet therapy was taken in about half of the patients suffering from intracranial hemorrhage.

**Table 3:** Anticoagulation and outcome in LVAD patients with late CVE.

Characteristics	Ischemic CVE (*n* = 23)	Hemorrhagic CVE (*n* = 13)
Anticoagulation at the time of stroke		
Phenprocoumon	18 (78.3%)	6 (46.2%)
Phenprocoumon derangement		
intratherapeutic	11 (61.1%)	2 (33.3%)
subtherapeutic	3 (16.7%)	0
Above target	4 (22.2%)	4 (66.7%)
Antiplatelet therapy	15 (65.2%)	7 (53.8%)
Heparin	3 (13%)	7 (53.8%)
Argatroban	2 (8.7%)	0
NIHSS at the time of stroke	3 (IQR 3–12)	9 (IQR 2–40)
Outcome after stroke		
mRS	3 (IQR 2–5)	5 (IQR 2–6)
NIHSS	3 (IQR 0–17)	7 (IQR 1–40)
Mortality	7 (30.4%)	3 (23.1%)

Anticoagulation regimes at the time of stroke that occurred at a later stage. The outcome after stroke was assessed at the time of discharge after stroke. In five cases, incidentally detected stroke signs were found in the imaging, which occurred after LVAD implantation, not corresponding to acute neurological symptoms and thus being considered silent strokes. However, these silent strokes are not assigned to an appropriate type of anticoagulation in Table 3, nor in further analysis, due to the missing onset with corresponding laboratory values. As indicated, data are presented as numbers (%) or median (IQR). CVE, cerebrovascular event; mRS, modified rankin scale; NIHSS, National Institutes of Health Stroke Scale.

### Recanalizing therapies

Intravenous thrombolysis (IVT) was administered as an acute thrombolytic agent in one patient with a large vessel occlusion of the anterior circulation, revealing a subtherapeutic INR range. This patient died as a consequence of a severe ICH following endovascular treatment. Mechanical thrombectomy was performed in a total of five patients with large vessel occlusion in the anterior circulation (*n* = 4) and one basilar artery occlusion. In four cases, postoperative hemorrhages and, in one case, a peri-interventional dissection occurred. All patients had poor outcomes, and in addition to the patient mentioned above, a second person died due to space-occupying intracranial edema (NIHSS at discharge: 12,17,17; mRS: 3,4,5).

### Stroke associated outcome

Patients with early CVE had a higher median NIHSS score at the time of the CVE (14; IQR 4.75–40) than those with late CVE (6; IQR 3–20.5). However, mRS at discharge did not differ between patients with intracranial hemorrhage compared to patients with ischemic events [median mRS after initial intracranial bleeding 5 (IQR 2–6) vs. ischemic events 4 (2.5–5), *p* = 0.535]. Statistically, mRS at the first outpatient visit after LVAD implantation did not differ between patients with and those without CVE [3 (IQR 2–5) vs. 3 (IQR2–4), *p* = 0.146].

### Mortality

**Figure 4:**
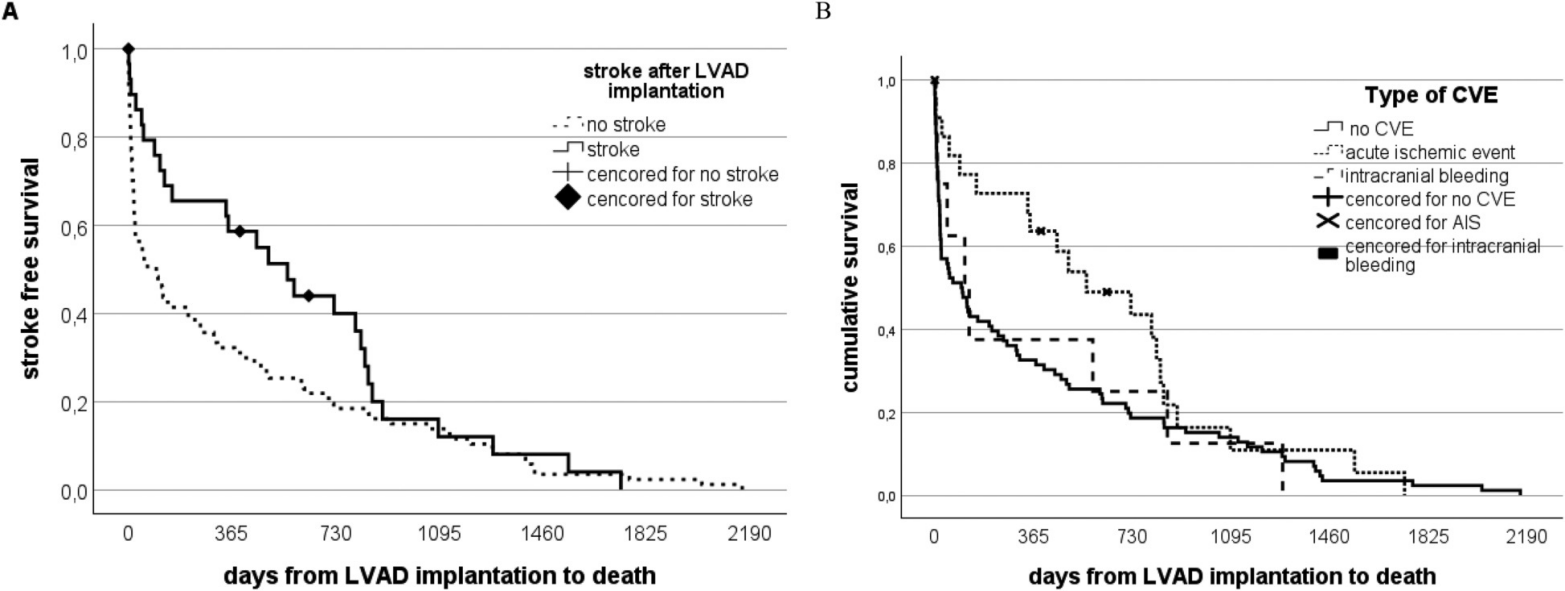
Stroke-free survival and stroke-free intervals in patients on LVAD therapy. **(A)** Patients on LVAD therapy are distributed according to the occurrence of stroke vs. a stroke-free long run. The solid black line represents the stroke group, while the dotted line represents the non-stroke group of the whole study cohort. The mortality rate in the stroke group is higher within the first 2 years compared to the non-stroke group. However, no significant difference is notable in the long run. **(B)** Patients on LVAD therapy distributed according to the type of stroke vs. a stroke-free long run. The solid black line represents all patients who did not suffer from stroke dying from other reasons than stroke. In contrast, the dotted lines represent patients with initially recorded intracranial bleeding compared to those with ischemic events.

Overall mortality did not differ between patients with and without CVE (*n* = 27/54; 50% vs. 94/195; 48.2%; *p* = 0.183) (Figure 4A). However, a significant proportion of patients died early for reasons other than stroke. Death was strongly related to the pre-interventional INTERMACS group, with better survival of patients with higher INTERMACS categories (test statistic 16.738720, *p* = 0.0002). Seven patients died as a direct consequence of stroke (early CVE *n* = 3; late CVE *n* = 4). Initial intracranial bleeding—irrespective of early or late—tended to be associated with worse survival (survival in intracranial hemorrhage: 120 days (IQR 19–792 days) vs. ischemic events: 495 days (IQR 93–835 days) *p* = 0.102 (Figure 4B).

## Discussion

In this study, the incidence and clinical course of stroke events in LVAD patients were assessed and systematically followed up at a high volume heart failure center. In contrast to registry-based stroke reports, this study focused on a detailed review of all available clinical documentation, including imaging data indicating a new-onset of CVE. According to our definitions, our main findings were the detection of two distinct clusters of increased stroke incidence: an early intra- and postoperative stage and another emerging in the later stage of LVAD support.

Additionally, we detected silent strokes in cerebral imaging during long-term follow-up in more than 5% of the study population with undeterminable onset and thus unclear association to LVAD therapy but also apparent occurrence after LVAD implantation compared to reference imaging.

The causes of CVE are complex in both early and late onsets, and identifying vulnerable patients remains difficult (33, 34). As risk factors for CVE during or directly after LVAD implantation, we identified the postoperative need for dialysis, whereas preceding kidney failure and low INTERMACS categories did not impact CVE occurrence. We found perioperatively identified cardiac thrombotic material to interfere with early CVE and observed that early stroke patients often showed a postoperative septic status.

Cho and colleagues identified four potential risk factors for ischemic stroke after LVAD implantation in the perioperative phase: perioperative ischemic or hypoxic events, pump thrombosis, inadequate antithrombotic therapy, and acute sepsis (29) resulting in pathophysiological mechanisms triggering inflammation and hypercoagulability. It is furthermore hypothesized that post-surgical inflammation promotes a pro-coagulatory status (35, 36). In accordance with these data, half of the strokes in our cohort occurred during the peri- and postoperative phases, supporting the hypothesis of a hypercoagulable and vulnerable period.

We observed that the need for dialysis following LVAD implantation was significantly linked to an increased risk of stroke occurrence in our study population. Acute kidney failure may be accompanied by a complicated postoperative course, including sepsis and multiorgan failure, ultimately contributing to major cardiovascular events (MACE). Correspondingly, a rapid decline in estimated glomerular filtration rate (37), the interplay of vascular comorbidities (38) and the dialysis treatment itself (39) may all contribute to stroke occurrence.

According to Dalia and colleagues, terminal chronic kidney failure requiring dialysis was associated with significantly higher one-year mortality compared to chronic kidney disease stages 3–5 in patients under LVAD therapy (40). Accordingly, requiring dialysis has become one of the most important contraindications for LVAD implantation in the United States (41). However, we found no increased stroke risk in the case of preoperative dialysis requirement, being in line with Dalias’ analysis, which revealed no significant difference in secondary outcomes such as bleeding, stroke, sepsis, or infection in the case of end-stage renal disease.

Systemic hyper- and hypocoagulable states are linked to postoperative infections and sepsis, driven mainly by device-related infections, as indicated by prior research (34). In our retrospective study, approximately one-third of the patients with early stroke met the criteria for sepsis, with pneumonia being the primary underlying cause.

Recent studies have shown that there is a higher risk of thromboembolism in antiplatelet non-responders, especially with HeartWare HVAD (42), and that a consecutive adjustment of antiplatelet therapy may reduce late-onset bleeding in LVAD patients (43). However, an aggregometry test is an error-prone and time-consuming procedure. It is, therefore, not yet part of a routine screening in LVAD centers, which is why this data is not available in our population.

Apart from systemic causes, the process of LVAD implantation and the device itself are known to trigger the formation of blood clots.

The surgery itself may have originated air, atherosclerotic or thrombotic embolisms, especially in the course of the outgraft connection to the aorta. But, these confounders are challenging to detect and need to be considered as potential biases of our analysis. Surgery-associated strokes and the cannula's axis position (20) represent important intraoperative contributors. Here, the LVAD material and its surface play a critical role by activating the extrinsic coagulation pathway and promoting a procoagulatory state through shear forces and blood stasis (44). The increased risk during this period suggests that surgical manipulation, vascular changes, or blood stasis during the surgical procedure may contribute to thrombus formation. The technological background is crucial in stroke genesis: HeartWare and HeartMate 3 use magnetically levitated centrifugal-flow pumps, whereas HeartMate II employs an axial pump. The improved blood flow in centrifugal-flow LVADs is linked to better outcomes and higher overall survival rates at 5 years compared to axial-flow LVADs (13). Thus, the low prevalence of acute ischemic stroke in the analysis mentioned above (29) appears remarkable. In June 2021, Medtronic stopped the distribution of the HeartWare HVAD due to a higher frequency of neurological adverse events and higher mortality associated with the device (45). In our study, we could confirm a trend toward the risk for early CVE in HeartWare patients but also found a relatively high stroke rate in HeartMate 3 patients. Compared to the reported stroke rate in HeartMate 3 patients by the AHA, our stroke rate of 0.15 events per patient-years appears high (46). But, apart from a different study design, the examined population of the MOMENTUM 3 study differs essentially from our population regarding the baseline characteristics, particularly concerning the INTERMACS levels.

Late CVE occurring after discharge from the index hospitalization were observed in 12.5% of our study population. Plecash et al. revealed cardiovascular risk factors such as arterial hypertension, atrial fibrillation (AF), diabetes mellitus, previous stroke, myocardial infarction, and smoking as the most critical contributors (33). Our results were consistent with most of the reported cerebrovascular risk factors, demonstrating that the occurrence of late stroke was significantly associated with a prior history of stroke before LVAD implantation. Additionally, several cardiovascular risk factors revealed a strong trend toward the increased risk of later strokes.

In both cardiac and non-cardiac interventions, the increased risk of stroke, particularly for patients with previous CVE, has already been recognized regardless of the time between ischemic stroke and surgery. Jørgensen and colleagues reported an adjusted 1.8–4.8-fold increased relative risk of 30-day mortality and 30-day MACE, respectively, in patients with a history of stroke compared to those without prior stroke (47).

Further cardiovascular risk factors have recently been identified as contributors to the risk of stroke, including AF. In a study by Stulak et al., of 389 patients who received an LVAD, those with preoperative AF had lower freedom from thromboembolic events after LVAD implantation (48). The lack of significance in our study might be attributable to a relatively small sample size.

We also made a counterintuitive observation of a negative correlation between the occurrence of strokes in patients with INTERMACS levels four to seven and patients who had stable cardiac conditions before LVAD implantation. This observation is not only due to a survival bias but also based on a collider bias (Berkson paradox), as our study design has been conditioned on hospitalization. Correspondingly, 39% of our observed late CVE occurred during current hospitalization. Reasons for hospitalization were various but most frequently LVAD-associated, pointing to a complex homeostatic interaction and an increased risk for imbalance of the hemorrhagic system.

Regarding management-related factors, we observed that strokes occurred during hospitalization while on bridging therapy with heparin. Thereby, the transition from VKA to heparin might be a particularly high-risk stage, at least for cerebral hemorrhages. However, the study design did not allow for drawing any evidence-based conclusions regarding the anticoagulation regimen and the cause of stroke. But the question remains of whether anticoagulation should not be handled more restrictively in LVAD patients—a discourse that has already been addressed in the LVAD community (49). The double-blind, placebo-controlled Aries study, which looked at the antiplatelet management in HeartMate 3 patients, represents an important step toward more personalized approaches in LVAD patients and demonstrates that a continuous re-evaluation of our therapeutic strategy is essential.

Notably, subarachnoid hemorrhages were more frequently reported in the literature during the postoperative course (34). Only a few patients suffered postoperative intracranial hemorrhages, according to our observation, also tending to represent a minority over the long term. However, increased susceptibility to hemorrhage is known to be complex while on MCS, knowing that causes of bleeding can be of ex- or endogenous nature. It has been described that shear forces and altered blood flow caused by devices can impair cerebral autoregulation and disrupt endothelial function (44). In addition, an acquired von Willebrand syndrome caused by advanced cardiac disease and shear forces of the devices leads additionally to a risk for hemorrhages (50). After considering numerous pathophysiological mechanisms, looking at the patients’ outcomes is highly relevant. It is noteworthy that patients suffering early strokes did not present any functional difference at the time of the first follow-up visit. In contrast, two-thirds of the patients with late CVE were discharged with a mRS of 3 or higher after administration for CVE (*n* = 18; 43.9%), indicating functional impairment following late CVE. Our study did not find a statistically significant difference in overall mortality between patients with and without CVE, and only a few patients died as a direct consequence of stroke. However, in addition to clinical decline, stroke-associated fears, and psychological stress, CVE are known to lead to poor outcomes (5, 8). Therefore, specialized interdisciplinary teams in a well-equipped hospital, experienced in treating a large number of LVAD and stroke patients, should continuously record, re-evaluate, analyze, and discuss prevention strategies to reduce stroke prevalence.

## Limitations

Due to the nature of the retrospective study design, it cannot be excluded that cerebrovascular events such as unreported TIA or silent strokes might have been unnoticed. The lack of frequent neurological co-evaluation and missing standardized head imaging at predefined visitations lead to a distorted stroke projection in our cohort due to a “confounding by indication” bias of head imaging. It is also essential to consider that in LVAD patients, magnetic resonance imaging of the head is not feasible, particularly impacting the detection of more minor, silent, or not yet properly demarcated strokes. Furthermore, analysis of clinical data revealed a small number of strokes, which were not distinctly attributed to a post-LVAD onset and declared as an indeterminate stroke. This might also result from missing standardized cerebral imaging and neurological assessment before LVAD implantation, mainly because patients’ conditions might have limited further assessments. However, undetected symptomatic strokes should be a minority, seeing that the clinical and functional impact of cerebrovascular events might have led to emergency evaluation and subsequent clinical and imaging diagnostics. Finally, follow-up periods differed individually, with extended follow-up periods for patients implanted in 2015 compared to later implanted patients. To solve this issue, we raised the claim to monitor all patients for at least 2 years minimum. However, due to a high mortality, the prevalence of late-onset CVE is not as representable as that of early strokes. By using competing risks analysis, we aimed to address these circumstances accordingly.

## Conclusion

Stroke remains a feared complication in LVAD patients. Our study demonstrates early and late CVE and points to an eminent proportion of unrecognized ischemic strokes, leading to underestimated rates of LVAD-associated complications.

In particular, the peri- and postoperative phases proved to remain a vulnerable moment. The high incidence of CVE and its association with poor outcomes, underscores the need for further awareness, investigations, and technical progress. Well-known modifiable risk factors for CVE must also be adequately addressed. Therefore, an interdisciplinary approach is needed to successfully minimize the risks of thromboembolic and hemorrhagic events in LVAD patients.

## Data Availability

The raw data supporting the conclusions of this article will be made available by the authors, without undue reservation.
