# Supplementary material for: Stroke in patients with left ventricular assist device (LVAD): who is at risk?—a retrospective observational study at a tertiary care center
Source: Front Cardiovasc Med. 2025 Sep 9;12:1591208. doi: 10.3389/fcvm.2025.1591208 (PMC12454346; doi:10.3389/fcvm.2025.1591208)
Supplement: Supplementary file 1 [file Datasheet1.pdf]

Supplementary Material

Material and Methods

Intracranial hemorrhage was confirmed when registering acute headache or new neurological deficiency, and CT scans revealed hyperdensities presenting hematoma or classical signs of intracranial bleeding, respectively. We also recorded transient ischemic attacks (TIA), defined as short neurological deficiencies lasting less than 24 hours with a lack of stroke-related signs in head imaging

Stroke was rated as “disabling” when mRS was > 3 at discharge after the event.

“Early stroke” was defined as any cerebrovascular event duringa hospitalization-related timeframe instead of a predetermined fixed time interval based on the complex and individualized interactions of pre-existing conditions, inflammatory response, coagulation status, and surface contact effects, all of which can vary and differently influence coagulation over the initial postoperative days and potentially extend over 30 days.
